# Potential antidepressant effects of a dietary supplement from Huáng qí and its complex in aged senescence-accelerated mouse prone-8 mice

**DOI:** 10.3389/fnut.2023.1235780

**Published:** 2023-07-28

**Authors:** Ming-Yu Chou, Yue-Ching Wong, Shih-Yi Wang, Ching-Hsin Chi, Teng-Hsu Wang, Mao-Jung Huang, Ping-Hsiu Huang, Po-Hsien Li, Ming-Fu Wang

**Affiliations:** ^1^School of Business, Qanzhou Vocational and Technical University, Jinjiang, China; ^2^International Aging Industry Research & Development Center (AIC), Providence University, Taichung, Taiwan (R.O.C.); ^3^Department of Nutrition, Chung Shan Medical University, Taichung, Taiwan (R.O.C.); ^4^PhytoHealth Corporation, Taipei city, Taiwan (R.O.C.); ^5^School of General Education, Hsiuping University of Science and Technology, Taichung, Taiwan (R.O.C.); ^6^School of Food, Jiangsu Food and Pharmaceutical Science College, Huai’an, China; ^7^Department of Food and Nutrition, Providence University, Taichung, Taiwan (R.O.C.)

**Keywords:** senescence-accelerated mouse prone-8, anti-aging, functional food, Huáng qí, anti-depressant

## Abstract

Healthcare is an emerging industry with significant market potential in the 21st century. Therefore, this study aimed to evaluate the benefits of tube feeding Huáng qí and its complexes for 8 weeks on 3-month-old senescence-accelerated mouse prone-8 (SAMP8) mice, 48 in total, randomly divided into 3 groups including control, Huáng qí extract [820 mg/kg Body weight (BW)/day], and Huáng qí complexes (6.2 mL /kg BW/day), where each group consisted of males (*n* = 8) and females (*n* = 8). Behavioral tests (locomotion test and aging score assessment on week 6, the single-trial passive avoidance test on week 7, and the active shuttle avoidance test on week 8) were conducted to evaluate the ability of the mice to learn and remember. In addition, after sacrificing the animals, the blood and organs were measured for antioxidant and aging bioactivities, including malondialdehyde (MDA) content and superoxide dismutase (SOD) activity and catalase activities (CAT), and the effects on promoting aging in SAMP8 mice were investigated. The findings showed that Huáng qí enhanced locomotor performance and had anti-aging effects, with positive effects on health, learning, and memory in SAMP-8 mice (*p* < 0.05), whether applied as a single agent (820 mg/kg BW/day) or as a complex (6.2 mL/kg BW/day) (*p* < 0.05). Based on existing strengths, a more compelling platform for clinical validation of human clinical evidence will be established to enhance the development and value-added of astragalus-related products while meeting the diversified needs of the functional food market.

## Highlights

– The benefits of Huáng qí did not vary by mouse gender.
– The physiological status of the mice was not affected by Huáng qí.
– The antioxidant activity of the Huáng qí complex was superior to Huáng qí *in vivo*.
– Huáng qí enhanced locomotion and had anti-fatiguing, and antiaging effects in mice.
– Huáng qí increased antioxidant activity and minimized oxidative damage in mice.

## 1. Introduction

All organs and physiological functions are affected by aging, notably leading to a physical decline in the elderly with a high risk of malnutrition, infirmity, morbidity, and mortality (1–5). Several studies have reported that aging-induced loss of protein balance leads to a loss of muscle mass, which affects the functioning of the gastrointestinal tract, thus reducing nutrient absorption (3, 6). In addition, psychological and social factors lead to inadequate absorption of nutrients (7). However, as the most significant risk factor for all diseases, aging seriously challenges global social resources and health insurance systems. The growth rate of the elderly population over 60 years has been significantly higher than that of younger people (8–10), and the World Health Organization predicted a doubling in the proportion of the world’s population over 60 years old by 2050 (2). Thus, healthy aging has become a concern despite the biological definition of aging as a progressive accumulation of diminished or complete loss of function (11). However, age-related phenomena, such as decreased immune system function, chronic inflammation, and disturbed gut microbiota, have also been extensively studied, with proper nutritional supplementation proven to improve or delay the onset, as food nutrition is one of the pillars of health (2, 4, 12–15).

Unfortunately, there are limitations of the approved and available medications (provide symptom relief rather than prevent or improve disease progression) (16). More recently, the role of dietary interventions or nutritional supplements to retard aging has become the focus (17–19). Therefore, it is important to address healthy aging and propose potential therapeutic approaches to limit aging-initiated disease and progression (5, 16, 20, 21). Several studies have been performed to repeatedly demonstrate the brain’s vulnerability to the harmful effects of increased oxidative stress (OS), which also explains the increase in reactive oxygen species (ROS) and antioxidant defenses has been associated with brain structural changes, higher lipid content, rapid metabolic rate, and proinflammatory signaling pathways, thus contributing to the pathogenesis of depression (5, 19, 22–24). Consequently, identifying suitable natural sources of antioxidants to combat the overproduction of ROS and OS is an effective preventive strategy to ameliorate aging-related diseases.

The primary bioactive components in Huáng qí (the root of *Astragalus membranaceus* (Fisch.) Bge. var. mongholicus (Bge) Hsiao) are saponins, polysaccharides, and flavonoids (25–30). In China, Japan, Korea, and Central and Southeast Asia, Huáng qí is used as a tonic to treat hepatitis, nephritis, and diabetes or as a complementary therapy for cancer. The Huáng qí herbal supplement is used chiefly as an immune stimulant in the United States to protect against influenza and upper respiratory tract infections (29, 31–34). Specifically, it is traditionally used with other herbs (ginseng, atractylodes, tangerine peel, rhizoma cimicifugae, bupleurum, licorice, and angelica) to enhance the immune system (31, 34–36). Interestingly, Huáng qí (animal mode) has been reported to have immunological functions that stimulate and alleviate depression while avoiding the effects of toxins (35, 37, 38). Moreover, in the cellular mode (Caco2 cell line), *Astragalus* polysaccharides (APS) have been modified by gamma irradiation to enhance immunomodulatory activity without changing the functional groups (only improved physicochemical properties) (39). In addition, APS enhances the activities of macrophages, natural killer cells, dendritic cells, T- and B- lymphocytes, and microglia while inducing the expression of various cytokines and chemokines (40–44). Notably, pharmacological findings suggest that components in a Huáng qí extract increase telomerase activity and antiaging effects (29, 34). Hence, we aimed to evaluate the effects of Huáng qí on learning and memory in the brain as well as antiaging ability in SAMP8 mice. We expected to develop a potential formulation to prevent aging-related diseases or a complementary therapy to regular therapy.

## 2. Materials and methods

### 2.1. Materials

Huáng qí (root of *A. membranaceus* (Fisch.) Bge. var. *mongholicus* (Bge) Hsiao) was provided by PhytoHealth Co. (Taipei, Taiwan). It was washed in distilled water, wiped dry, frozen at −20°C for 24 h, and freeze-dried. The powder was dry -milled in a homogenizer (through an 80-mesh sieve). Next, 3.5 L of 95% alcohol and 210 g of Huáng qí powder were placed in a 5 L flask. The flask was shaken every 4 h for 24 h. The alcohol was removed by rotary vacuum evaporation, followed by freeze -drying. The freeze-dried sample was labeled the “Huáng qí extract.” The Huáng qí complex formula was composed of the Huáng qí extract, red dates (*Ziziphus jujuba* Mill.), and *Acanthopanax* root [*Acanthopanax senticosus* (Rupr. Maxim.) Harms, which were mixed and flavored with anhydrous D-trehalose and pure water], were provided by PhytoHealth Co. All chemicals were purchased from Sigma-Aldrich® (Merck KGaA, Darmstadt, Germany) for direct use without any pre-treatment.

### 2.2. Experimental animal

A total of 48 (half male and half female) 3-month-old SAMP8 mice were purchased from the National Laboratory Animal Center (Taipei, Taiwan) and randomly distributed into control, Huáng qí extract, and Huáng qí complexes groups, each consisting of males (*n* = 8) and females (*n* = 8). SAMP8 mice were characterized by short life spans, lack of learning and memory, neuronal damage, and amyloid accumulation in the brain, indicating suitability for aging-related learning and memory studies. All mice were housed in plastic cages [30 (L) × 20 (W) × 10 (H) cm^3^] by gender (*n* = 8), and the room was maintained at a specific pathogen-free with temperature (25 ± 2°C) and humidity (65 ± 5%). At the same time, the dark period (07:00–19:00) and light period (19:00–07:00). In addition, feed (AIN-93 M standard purified feed) and water were supplied *ad libitum,* which were renewed every morning. All the mice were randomly grouped according to gender (*n* = 8) following one week of adaptation. All groups consisted of a control group, a Huáng qí extract (820 mg/kg BW/day) group, and Huáng qí complexes (6.2 mL/kg BW/day) group. All samples were dissolved in distilled water and tube fed to each mouse once a day for 8 weeks. Moreover, the doses were based on “Guidelines for the Testing of Chemicals method, Section 4 Test No. 488: Transgenic Rodent Somatic and Germ Cell Gene Mutation Assays” from the Organization of Economic Cooperation and Development (OECD)‘s recommended daily intake per kg BW of a 60 kg adult, namely the human equivalent dose, and then extended by a factor of 12.3 (45), i.e., one-fold for mice; the detailed calculations are as follows.

Huáng qí extract:


RecommendeddailyBWintakeperkgfora60kgadulthumanequivalentdose=4g60kgBW/day=66.7mg/kgBW/day

$$\begin{array}{c} 1\;folddoseformice=66.7\;\mathrm{mg}/\mathrm{kg}\;\mathrm{BW}/\mathrm{day}\times 12.3\\ =820\;\mathrm{mg}/\mathrm{kg}\;\mathrm{BW}/\mathrm{day} \end{array}$$

Huáng qí complexes:


ecommendeddailyBWintakeperkgfora60kgadulthumanequivalentdose=30mL60kgBW/day=0.5mL/kgBW/day

$$\begin{array}{c} 1\;folddoseformice=0.5\;\mathrm{mL}/\mathrm{kg}\;\mathrm{BW}/\mathrm{day}\times 12.3\\ =6.2\;\mathrm{mL}/\mathrm{kg}\;\mathrm{BW}/\mathrm{day} \end{array}$$

Finally, each BW of mice was used to calculate the daily sample tube feeding. Each group of BW, diet, and water intake changes were recorded weekly during the experiment. The locomotion and grading scores were evaluated at week 6, and the memory learning ability (passive avoidance task and active shuttle avoidance test) was assessed at weeks 7 and 8. Subsequently, mice were anesthetized with sodium pentobarbital (using a saline solution prepared to a concentration of 1% with a dose of 50 mg/kg), followed by cardiac blood harvesting and sacrifice (8 h fasting before surrender). Next, the serum biochemical analysis, brain, and organs were measured for bioactivity indicators related to aging, as detailed in the subsequent sections.

All animal procedures followed the standards outlined in the guidelines for the Care and Use of Experimental Animals by the Committee for the Purpose of Control and Supervision of Experiments on Animals and the National Institutes of Health. The Committee on Animal Research, Providence University, under code 220191211 A008 approved the protocol.

### 2.3. Grading score evaluation

The grading score was evaluated at week 6 and followed the grading score system by Takeda et al. (46). Each item had 5 levels according to the definition of each level, whereas high scores indicated that the mouse was aged seriously. Specifically, (I) Behavior: observe the reaction of mice to exploration (reactivity) within 30 s and the avoidance response (passivity) when the operator pinched the skin on the back of the neck. (II) Appearance: observe hair glossiness, coarseness, hair loss, and ulcers. (III) Eyes: observation of mucositis around the eyes and edema and redness of the eyelids (periophthalmic Lesions). (IV) Spine: observe the changes in the anterior and posterior curvature of the spine (spine lordokyphosis). The total score of each item was calculated.

### 2.4. Locomotor activity test

In this study, the locomotor activity test was performed as described by Weber et al. (47) with minor modifications. This evaluation was to observe the locomotion activity of mice in the local spatial plane. Briefly, a mouse was placed in the center of a 25 (L) × 25 (W) × 25 (H) cm^3^ aluminum chamber and operated under weak light and quiet environments, whereas data were collected by video-recorded (FDR-AX700, Sony, Tokyo, Japan) observations, including the amount of activity and activity (rest time, exercise time, horizontal and vertical movement) of each mouse moving in a plane for 10 min. In addition, the interior space of the chamber was cleaned with a water-soaked Kimwipe to minimize the effect of odor following the completion of each mouse.

### 2.5. Passive avoidance task

The evaluation methodology was based on Lin et al. (48) with slight modifications. This study was conducted in a 35 (L) × 17 (W) × 20 (H) cm^3^ shuttle cage (Model E10-15, Coulbourn Instruments, Massachusetts, USA). The inner part includes a light chamber and a dark chamber with a 7.5 (L) × 6.5 (W) cm^2^ guillotine door (Model E10-15GD, Coulbourn instruments) in the center, which allows access to each other, while the bottom of the chamber has spaced (1 cm) and parallel metal rods with electric current. The mouse was initially placed in the light chamber, followed by 10 s of acclimatization, and then the guillotine door would be opened, allowing free exploration between the two chambers. However, by nature, the mouse is nocturnal behavior in the dark and will move to dark places. Upon entering the dark chamber, the guillotine door would be closed quickly, followed by an aversive stimulus (such as a foot shock) of 0.5 s (0.5 microamperes) at 5-s intervals for three consecutive times during the training period. Subsequently, the memory capacity of the mice was tested again at 24 h, 48 h, and 72 h. It was performed in the same way as described above, but without giving any electric shock. Meanwhile, a mouse staying in the light chamber was recorded, and the maximum duration of each test was 180 s. Lengthier time spent in the light room means the mouse has better memory capacity.

### 2.6. Active shuttle avoidance test

The evaluation followed the Weber et al. (47) described and was modified as appropriate. In brief, the same device as station 2.5 was used. However, a different device was set up with lights and sound at the top of the shuttle cage, controlled by a computer to stimulate the mice. Initially, the mouse was placed in a chamber within 10 s at the beginning of the test. Subsequently, light and sound-conditioned stimulus (CS) with a 10-s interval was given. In case the mouse remained in the same chamber during the CS demonstration, an electric shock of 0.3 mA for 5 s was automatically delivered as an unconditioned stimulus (UCS). In contrast, no shock was delivered once the mouse entered another chamber during the CS demonstration. Moreover, there is also a balance device at the center bottom, which utilizes the principle of stilts by sensing the left or right side of the mouse’s current position in the cage. The computer controls the experimental process for the time, sound, light, and electric shock, which finally records the results. During the process, the mouse stays on the same side, which means that the mouse has not yet learned to deliver the electric shock, which is a training process for learning. Conversely, a mouse moving to the other side indicated it had known without giving an electric shock, referring to its memory performance. The computer automatically determines whether the mice respond to the conditioned stimulus CS or UCS through the above balance device. Each mouse undergoes 5 rounds of the same test at each 15–20 min interval for four consecutive days. Notably, more successful mouse evasions were associated with better learning memory.

### 2.7. Relative organs weight (%)

This study’s relative organs weight according to Chou et al. (49) described method. All SAMP8 mice were weighed for brain, heart, liver, spleen, lung, and kidney organs immediately after sacrifice while calculating relative organ weights according to the following formula. Simultaneously, each organ was evaluated with the naked eye, and any abnormalities such as abnormal color, enlargement, or hard masses were found, and further histopathological stations would be performed.


Relativeorgansweight(%)=OrganweightBW×100

### 2.8. Serum biochemical parameters analysis

Serum biochemical parameters analysis in this study was performed based on the approach described by Chou et al. (12). Blood from the mice, as mentioned earlier, obtained by cardiac blood collection was immediately centrifuged at 4°C and 12,000 × g for 10 min using a Microfuge 22R (Beckman Coulter Inc., Brea, Calif., USA). The analysis of plasma biochemical parameters included glucose, total protein, albumin, triglycerides, total cholesterol, high-density lipoprotein cholesterol (HDL-C), low-density lipoprotein cholesterol (LDL-C), glutamate-oxalate transaminase (GOT), glutamate pyruvate transaminase (GPT), blood urea nitrogen (BUN) and creatinine. The operation followed the manufacturer’s specification with the Synchroh LX-20 system (Beckman Coulter Inc., Brea, Calif., USA).

### 2.9. Determination of the bioactivity indicators in brain and liver tissues

#### 2.9.1. Determination of MDA content

##### 2.9.1.1. Preparation of brain tissue

Whole brain tissue extraction was performed according to Chou et al. (12) and Lee et al. (13) described methods. Briefly, whole brain tissue was homogenized (for 30 s at 1,400 rpm in an ice bath) by adding 1 mL of 50 mM PBS (pH 7.4) using a Polytron^®^ homogenizer (PT 3000, Thomas Scientific LLC, Swedesboro, NJ, USA). Next, remove unbroken tissue and debris at 15,000 × *g* for 30 min at 4°C via a centrifuge (HermLe Z383K, Benchmark Scientific Inc. Sayreville, NJ, USA). At the same time, the obtained supernatant was analyzed for MDA content as described in section 2.9.1.3.

##### 2.9.1.2. Preparation of liver tissue

The collected mouse liver was cut 0.1 g, added to 2,000 μL of 50 mM sodium phosphate buffer (PBS, pH 7.0), and homogenized for 30 s at 1,400 rpm in an ice bath using a Polytron® homogenizer (PT 3000), while to the preparation of the liver tissue homogenization solution was for MDA content determination as described in next section.

##### 2.9.1.3. Measurement of MDA content by the thiobarbituric acid reactive substances (TBARS) method

MDA content was determined according to the description of Ornoy et al. (50) with modifications. 150 μL of the above sample (in 2.9.1.1) was added with 300 μL of 2-thiobarbituric acid (TBA) colorant and 45 μL of butylated hydroxytoluene (BHT) solution, vortexed for 1 min, and reacted in a water bath at 90°C for 45 min. Afterward, the solution was cooled at room temperature, extracted with n-butanol (vortex shaking for 1 min), and centrifuged at 1,000 × *g* for 5 min at 4°C using a centrifuge (HermLe Z383K, Benchmark Scientific Inc). Finally, the absorbance value was measured with a UV/Vis spectrophotometer (DU530, Beckman coulter Inc., Brea, CA, USA) at wavelength 535 nm. In addition, standard curves were prepared using known concentrations (0.625, 1.25, 2.5, 5, 10, and 20 μM) of MDA, which were interpolated to calculate the sample MDA concentration (mM/g tissue).

#### 2.9.2. Determination of SOD

This study used the SOD (Ransod) assay kit (SD125, Randox Laboratories Ltd., Moorgate, UK) as described in its protocol. It works on the xanthine oxidase (XO) principle to produce superoxide anion (O_2_^-•^), followed by a reaction with 2-(4-iodophenyl)-3-(4-nitrophenol)-5-phenyltetrazolium chloride (I.N.T.) to make red formazan dye. One unit of SOD was used to produce a 50% inhibition of the reduction rate of I.N.T. under the assay conditions. Finally, the activity of SOD was measured by the degree of such reaction inhibition.

Specifically, 0.1 mL of liver tissue solution (as in 2.8.1) was mixed with 400 μL of cold deionized water and centrifuged at 4°C for 15 min. Then, 10 μL of supernatant was mixed with 490 μL of sample diluent solution (R1b Buffer), and the formazan dye content was determined using a spectrophotometer at a wavelength of 340 nm. The standard curve was prepared by serial dilution with the standard included in the kit using the same procedure described above. The SOD activity (U/g tissue) of the samples was calculated by interpolation.

#### 2.9.3. Determination of CAT activity

This study determined the CAT activity by CAT peroxidation using an assay kit (Item No. 707002, Cayman Chem). The CAT will produce formaldehyde with methanol in the appropriate concentration of H_2_O_2_, whereby Purpald (4-amino-3-hydrazino-5-mercapto-1,2,4-triazole) with trans-formaldehyde formed a bicyclic heterocyclic structure with a colorless to purple color after oxidation, which measured the activity of CAT at wavelength 540 nm. 1 nmol of H_2_O_2_ degradation per min per g of tissue catalyzed was defined as 1 CAT activity unit (U).

Briefly, in a 96-well dish, 20 μL liver tissue solution (as in 2.8.1) was added to one well, followed by 100 μL assay buffer and 30 μL methanol. Next, 20 μL of hydrogen peroxide was rapidly added to initiate the reaction with 20 min (25°C) incubation on an oscillator followed by 30 μL of potassium hydroxide to terminate the reaction and subsequently 30 μL of Purpald reaction for 10 min (25°C). Finally, 10 μL potassium hydroxide was added and incubated on an oscillator for 5 min, followed by measurement of the absorbance at 540 nm. The standard and positive control operated in the same way. After preparing the standard curve, the sample’s formaldehyde (μM) content was calculated by interpolation. Finally, the CAT activity of the sample was calculated by the following formula.


$$\begin{array}{c} \mathrm{CAT}\;\mathrm{activity}\;\left(U/g\;\mathrm{tissue}\right)=\frac{sample^{\prime }s\;formaldehyde\;\left(\mu M\right)\;content\;}{20\;\min}\\ \times \;\;Sampledilutionratio \end{array}$$

### 2.10. Statistical analysis

All data were expressed as mean ± standard error (SEM), while all measurements were performed in triplicate with statistical analysis by IBM SPSS Statistics (version 22, IBM Corp., Armonk, N.Y., USA).

The differences between groups were examined using a one-way analysis of variance (ANOVA). Duncan’s multiple allometric tests were used to compare the differences between groups, while the experimental results represented a significant difference (*p* < 0.05).

## 3. Results and discussion

### 3.1. BW variations, average diet, and water intake comparison

The overweight condition is positively associated with the risk of chronic disease and death, while long-term high BW, overweight, or obesity has been associated with the shortening of telomeres in the middle-aged and elderly populations (51, 52). Obesity has been linked to depleted leukocyte telomere length by other non-inflammatory mechanisms (53). Obesity and inflammation are associated with aging (51, 53). The BW changes, average diet, and water intake in the male and female groups (*n* = 8) of SAMP8 mice fed Huáng qí and the Huáng qí complex for 8 consecutive weeks (Table 1) were consistent, and no significant differences were observed between the groups. The mice were randomly grouped at trial entry with no significant difference in the initial BW; therefore, both Huáng qí (820 mg/kg BW/day) and the Huáng qí complex (6.2 mL/kg BW/day) had no adverse effects on the physiology, growth, or development of the mice. Notably, Pačesová et al. (54) reported the SAMP8 mice gradually gain weight from month two onwards, which is evidence of the aging process, where the trend in weight change was consistent with our study, which used 3-month-old SAMP8 mice at the beginning of the study. Furthermore, pathological features (e.g., decreased number of neurons and increased neuroinflammation) were reported in the published literature in the SAMP8 mice beginning at three months of age (54).

**Table 1:** (1) Weight change, diet, and water intake; (2) grading score evaluation; (3) locomotor evaluation; (4) relative organs weight for the SAMP8 mice groups (*n* = 8) during the experimental period.

Genders		Male			Female		
Group (*n* = 8)		Control	Huáng qí (820 mg/kg BW/day)	Huáng qí complexes (6.2 mL/kg BW/day)	Control	Huáng qí (820 mg/kg BW/day)	Huáng qí complexes (6.2 mL/kg BW/day)
BW (g)	Initial	28.16 ± 0.44^a^	28.09 ± 0.19^a^	27.80 ± 0.20^a^	28.34 ± 0.26^a^	28.86 ± 0.30^a^	28.52 ± 0.34^a^
	Final	29.85 ± 0.37^a^	29.95 ± 0.35^a^	29.80 ± 0.26^a^	29.03 ± 0.35^a^	29.48 ± 0.37^a^	28.81 ± 0.26^a^
Gain/BW		1.70 ± 0.34^c^	1.85 ± 0.26^c^	2.00 ± 0.31^d^	0.69 ± 0.15^b^	0.62 ± 0.12^b^	0.29 ± 0.13^a^
Intake	Diet (g/day)	5.34 ± 0.04^a^	5.43 ± 0.04^a^	5.33 ± 0.03^a^	5.03 ± 0.08^a^	4.99 ± 0.11^a^	5.05 ± 0.07^a^
	Water (mL/day)	5.39 ± 0.03^a^	5.35 ± 0.06^a^	5.42 ± 0.02^a^	5.04 ± 0.19^a^	5.03 ± 0.17^a^	5.06 ± 0.17^a^
Grading score							
Behavior	Reactivity	1.25 ± 0.16	1.00 ± 0.19	1.00 ± 0.19	1.38 ± 0.32	1.13 ± 0.23	0.75 ± 0.25
	Passivity	1.38 ± 0.26	1.25 ± 0.16	0.88 ± 0.13	1.13 ± 0.35	1.00 ± 0.33	0.50 ± 0.19
Skin	Glossiness	1.00 ± 0.19	1.00 ± 0.19	0.88 ± 0.13	1.25 ± 0.25	1.13 ± 0.30	0.88 ± 0.30
	Coarseness	1.25 ± 0.16	1.25 ± 0.16	0.88 ± 0.23	1.38 ± 0.26	1.13 ± 0.30	0.75 ± 0.25
	Hair loss	1.38 ± 0.18	1.38 ± 0.18	1.25 ± 0.16	1.63 ± 0.18	1.13 ± 0.23	1.00 ± 0.27
	Ulcer	0.25 ± 0.16	0.13 ± 0.13	0.13 ± 0.13	0.13 ± 0.13	0.25 ± 0.16	0.25 ± 0.16
Eyes	Periophthalmic lesion	1.13 ± 0.13	1.25 ± 0.16	1.13 ± 0.23	1.38 ± 0.18	1.00 ± 0.27	0.63 ± 0.18
Spine	Lordokyphosis	0.50 ± 0.19	0.50 ± 0.19	0.38 ± 0.18	0.63 ± 0.18	0.50 ± 0.19	0.75 ± 0.16
Total		8.13 ± 0.55^a^	7.75 ± 0.37^ab^	6.50 ± 0.42^b^	8.88 ± 1.19^a^	7.25 ± 0.67^ab^	5.50 ± 0.63^b^
Locomotion (s/5 min)							
0–5		112.38 ± 1.70 ^a^	107.75 ± 1.57^a^	109.88 ± 1.61^a^	116.25 ± 1.88^a^	105.75 ± 1.52^a^	111.13 ± 1.87^a^
6–10		82.38 ± 1.83^C^	76.38 ± 1.25^b^	76.50 ± 1.89^b^	71.25 ± 1.81^ab^	68.75 ± 1.51^a^	75.00 ± 1.85^b^
Relative organ weights (g/100 g BW)							
Brain		1.44 ± 0.06^a^	1.43 ± 0.03^a^	1.51 ± 0.03^a^	1.58 ± 0.03^a^	1.50 ± 0.04^a^	1.59 ± 0.03^a^
Heart		0.52 ± 0.04^a^	0.54 ± 0.04^a^	0.56 ± 0.03^a^	0.47 ± 0.03^a^	0.52 ± 0.03^a^	0.52 ± 0.03^a^
Liver		4.52 ± 0.29^a^	4.84 ± 0.38^a^	4.88 ± 0.23^a^	4.69 ± 0.22^a^	4.52 ± 0.08^a^	4.68 ± 0.13^a^
Spleen		0.35 ± 0.02^a^	0.38 ± 0.02^a^	0.38 ± 0.02^a^	0.36 ± 0.04^a^	0.37 ± 0.04^a^	0.32 ± 0.02^a^
Lung		0.63 ± 0.04^a^	0.66 ± 0.02^a^	0.69 ± 0.03^a^	0.67 ± 0.04^a^	0.62 ± 0.03^a^	0.64 ± 0.02^a^
kidney		1.54 ± 0.08^a^	1.72 ± 0.12^a^	1.77 ± 0.07^a^	1.74 ± 0.07^a^	1.73 ± 0.04^a^	1.69 ± 0.04^a^

Values are mean ± SEM. Different lowercase letters on each line indicate a significant difference (*p* < 0.05). BW, Body weight.

### 3.2. Evaluation of grading score (6th-week post-feeding)

Takeda et al. (46) developed a grading score system consisting of nine items scoring 0, 1, 2, 3, and 4 depending on the degree of aging. Finally, the total grading scores were used to evaluate the degree of aging in the senescence-accelerated mouse (SAM), where a higher score refers to more severe aging. In addition, SAMP8 mice developed normally but with rapid aging properties, such as hair loss and disturbances in day-night rhythm (55). In this study, male and female SAMP8 mice were fed Huáng qí and the Huáng qí complex for 6 consecutive weeks. The grading scores of the groups were evaluated, and the experimental group had a lower total grading score than the control group (Table 1). In particular, the total grading scores of the males and females fed the Huáng qí complex were significantly different (*p* < 0.05). Therefore, the grading score evaluation results indicate that providing the Huáng qí complex may reduce the age level of SAMP8 mice. Interestingly, the total grading score was better in females (5.50 ± 0.63) than in males (6.50 ± 0.42).

### 3.3. Locomotor (6th-week post-feeding)

Commonly known behavioral disorders, cognitive decline, psychiatric disorders, and dementia associated with depression are risk factors for AD (56–60). Therefore, deferring or minimizing these symptoms is essential to maintain the quality of life of the elderly (58). As a result, locomotion activity (sec/5 min) was not significantly different in the male and female groups of mice administered Huáng qí and its complex (Table 1). Moreover, locomotion decreased following the extended time during the 5 min post-test. This was attributed to the high locomotor activity of the initial exploratory response immediately after encountering an unfamiliar environment. However, as the mice acclimated to the environment, locomotor activity decreased, thus leading to a decrease in locomotion activity. Despite the decrease in locomotor activity in the SAMP8 mice, the effects of the Huáng qí complex were more pronounced. The potential mechanism of the antiaging effect of Huáng qí and its complex may be inhibiting OS in the brain. However, the mechanism remains unknown and warrants further study.

### 3.4. Learning and memory testing of the mice

Various studies have suggested that cognitive and motor deficits occur in aging rodents, apart from dysregulation of epigenetics during learning and memory processes, which has been associated with AD pathogenesis (61–63). The effect of epigenetic processes on learning and memory extends to the sensory system, where all core cue-evoking processes dependent on experience begin in the brain (64). Moreover, stress, fear, and anxiety-like behaviors have been associated with neurodegeneration, aging, and AD (62, 65, 66). Behavioral avoidance tests have been applied to assess learning and memory (48). Memory is defined as a change in behavior caused by experience, and learning is the process of memory acquisition achieved by engaging neurotransmission and neurons (24). The passive avoidance task and active shuttle avoidance test were performed on weeks 7 and 8 in the SAMP8 male and female mice fed Huáng qí and its complex to evaluate the effects on learning and memory. The passive avoidance task was based on the duration in the light chamber. The active shuttle avoidance test was based on the escape response of the mice.

#### 3.4.1. Passive avoidance task

**Figure 1:**
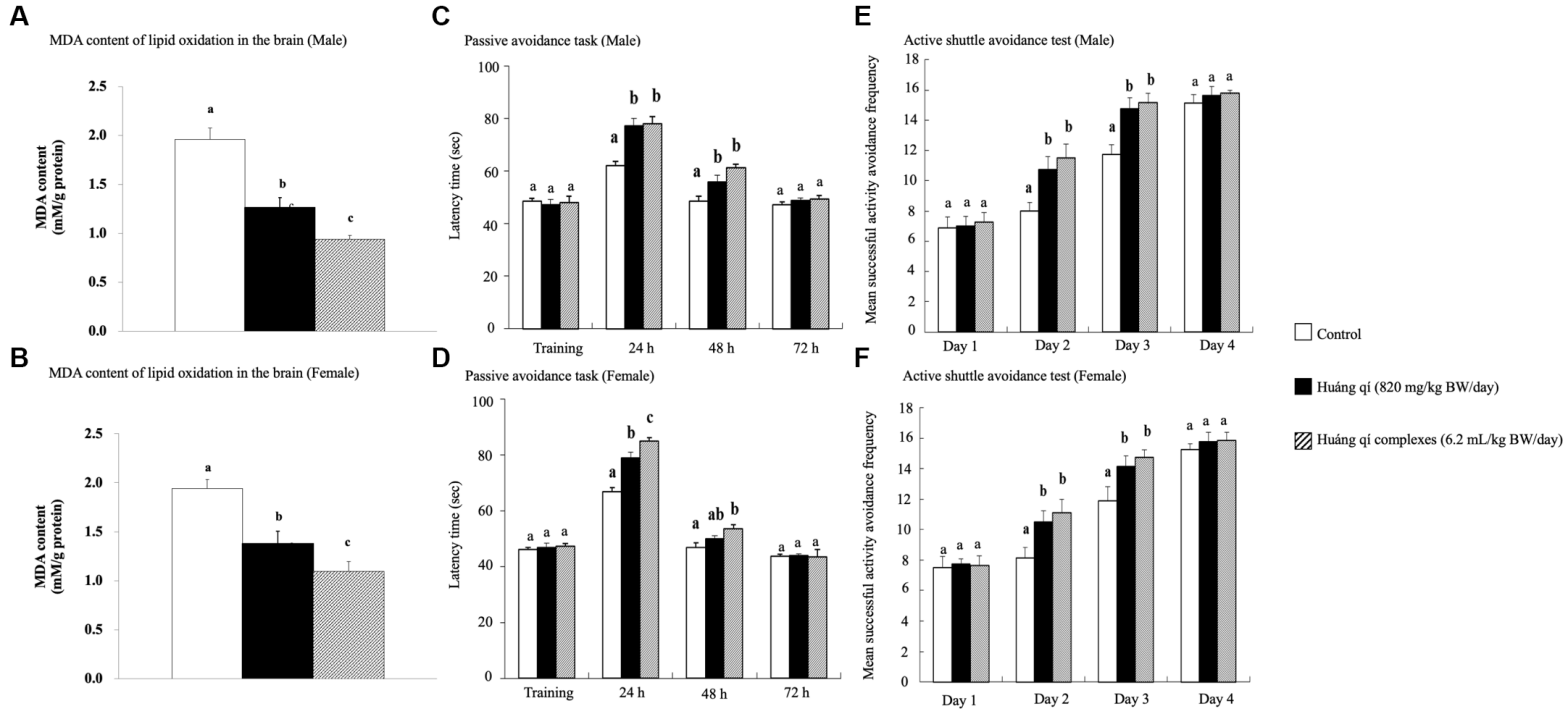
Effects of continuous 8-weeks of tube feeding Huáng qí and its complex on SAMP8 mice (*n* = 8) on **(A, B)** MDA content of lipid oxides in the brain; **(C, D)** the passive avoidance task; **(E, F)** the active shuttle avoidance test. Different lowercase letters represent significant differences (*p* < 0.05).

The passive avoidance task is a fear-driven test, and mice with better memory and learning avoided entering the hazardous areas (48). All SAMP8 mice were fed Huáng qí and its complex during week 7 for the passive avoidance task (Figures 1C,D). As results, no significant differences in the learning results of male mice were detected on the first day of training in any of the groups (Figure 1C). However, the time in the length chamber increased significantly compared to the control group 24 and 48 h after training in both experimental groups of male mice (*p* < 0.05). The training results in female mice on the first day were the same as in males, with no significant differences between the groups (Figure 1D). However, the female mice in both experimental groups remained in the light chamber significantly longer than the control group (*p* < 0.05) after 24 h of training, with a satisfactory effect in the Huáng qí group. The time remaining in the light chamber increased after 48 h of training in both experimental groups of female mice. Significantly more time was spent in the light chamber by Huáng qí group than in the control group (*p* < 0.05). Notably, no significant difference in the time remaining in the light chamber was detected for the male or female mice 72 h after-training, which may have been due to the electric shock given during training, which caused a longer retention time in the light chamber. Unfortunately, memory-retaining ability decreased with time, resulting in a decrease in the time spent in the light chamber by 72 h post-training. Notably, Lee et al. (13) reported the same findings for SAMP8 mice fed a diet supplemented with a liquid fermentation medium of *Hericium erinaceus* mycelium for 13 weeks; no improvement in learning performance was detected on the passive avoidance task training day, but improved learning and memory with the ability to retain this training memory were observed post-training.

#### 3.4.2. Active shuttle avoidance test

An experimental animal moving to another chamber immediately preceding the onset of the conditioned stimulus was considered active avoidance, whereas if the animal ran to the other side after receiving an electric shock, the reaction was considered an escape movement (67). The active shuttle avoidance test used a double-sided shuttle. The experiment animals moved immediately to the other side regardless of where the animal was placed when the sound began or the lights went out, indicating that neither path is better than the other (67–69). The active shuttle avoidance test was performed on week 8 while feeding the Huáng qí and its complex. The number of successful avoidance attempts was not significantly different between the groups of mice on day 1 (Figures 1E,F). This occurred because all mice were still in the learning stage. However, the experimental groups (fed Huáng qí and its complex) were significantly more avoidant on days 2 and 3 compared with the control group (*p* < 0.05). The difference in response between training and testing was evidence for generating new memories (68). Moreover, this study showed an increase in successful response avoidance on day four but no significant differences between the groups. Along with other similar studies, the mice in the experimental groups experienced significantly lower retention than the control group regarding the frequency of electroshock while improving memory (56, 70, 71). Hence, this study showed that supplementation with Huáng qí or its complex for 8 consecutive weeks promoted learning and memory in the mice.

### 3.5. Variations in relative organ weights

The relative organ weight is useful for identifying normal or abnormal organ weights (72). Brain weight has no relationship with BW but has a strong negative correlation with age (72). The weights of the brain, heart, liver, spleen, lungs, kidneys, and the organs in all experimental groups fed Huáng qí, and its complex for 8 weeks were not significantly different compared with the control group (Table 1). However, no abnormalities, such as enlargement or hard masses, were observed with the naked eye, and the color remained normal. Thus, providing Huáng qí to the mice did not affect the organs or cause any damage.

### 3.6. Serum biochemical parameters

The serum biochemical results were not significantly different between the experimental and control groups after continuously feeding Huáng qí and its complex to male and female mice for 8 weeks (Table 2). Therefore, continuously supplementing with Huáng qí for 8 weeks did not affect the mice’s serum biochemical parameters or physiology.

**Table 2:** Serum biochemical parameters in the SAMP8 mice groups (*n* = 8) during the experimental period.

Gender	Group (n = 8)	GOT	GPT	Total	Albumin	BUN	Creatinine	Total Cholesterol (mg/dl)	Triglyceride	HDL	LDL	Glucose
		(U/L)	(U/L)	Protein(mg/dl)	(g/dl)	(mg/dl)	(mg/dl)		(mg/dl)	(mg/dl)	(mg/dl)	(mg/dl)
Male	Control	113.50 ± 1.16^a^	50.00 ± 1.57^a^	6.91 ± 0.15^a^	3.75 ± 0.08^a^	30.88 ± 1.59^a^	0.25 ± 0.02^a^	149.00 ± 0.98^a^	114.38 ± 1.63^a^	49.63 ± 1.82^a^	9.50 ± 0.63^a^	148.88 ± 1.60^a^
	Huáng qí (820 mg/kg BW/day)	112.13 ± 1.44^a^	50.25 ± 1.56^a^	6.86 ± 0.08^a^	3.78 ± 0.09^a^	32.23 ± 1.29^a^	0.24 ± 0.02^a^	149.13 ± 1.76^a^	111.38 ± 1.66^a^	48.50 ± 1.20^a^	9.25 ± 0.67^a^	150.38 ± 1.12^a^
	Huáng qí complexes (6.2 mL/kg BW/day)	112.63 ± 1.41^a^	49.13 ± 1.94^a^	6.88 ± 0.14^a^	3.79 ± 0.10^a^	30.58 ± 1.11^a^	0.26 ± 0.02^a^	149.88 ± 1.30^a^	111.75 ± 1.82^a^	49.63 ± 1.31^a^	9.75 ± 0.70^a^	149.88 ± 1.57^a^
Female	Control	112.00 ± 1.31^a^	51.13 ± 1.57^a^	6.85 ± 0.12^a^	3.81 ± 0.07^a^	30.90 ± 1.18^a^	0.33 ± 0.03^a^	150.50 ± 1.22^a^	111.88 ± 1.75^a^	48.25 ± 1.21^a^	9.25 ± 0.45^a^	149.50 ± 1.31^a^
	Huáng qí (820 mg/kg BW/day)	114.75 ± 1.79^a^	51.13 ± 1.96^a^	6.89 ± 0.11^a^	3.78 ± 0.09^a^	30.88 ± 1.10^a^	0.29 ± 0.01^a^	151.75 ± 1.44^a^	109.38 ± 1.93^a^	49.25 ± 1.35^a^	9.13 ± 0.67^a^	150.50 ± 1.50^a^
	Huáng qí complexes (6.2 mL/kg BW/day)	112.75 ± 1.21^a^	51.63 ± 1.34^a^	6.86 ± 0.11^a^	3.91 ± 0.11^a^	31.19 ± 0.89^a^	0.31 ± 0.01^a^	151.50 ± 1.25^a^	108.75 ± 1.88^a^	47.50 ± 1.61^a^	9.38 ± 0.68^a^	149.75 ± 1.50^a^

Values are mean ± SEM. Different lowercase letters on each column indicate a significant difference (*p* < 0.05). BW, Body weight.

### 3.7. Aging-related liver indicators

#### 3.7.1. Malondialdehyde in the brain and liver

Malondialdehyde (MDA) is an oxidized lipid product with neurotoxic properties, which represents ROS production. Sustained oxidant damage initiates the JNK/ERK signaling pathways to induce apoptosis (5, 22, 73). However, the free radical theory of aging refers to the slow production of ROS as an inevitable consequence of life with accumulated oxidative damage to cell membrane proteins and lipids (56, 74, 75).

The brain MDA contents of male and female mice in all experimental groups decreased after 8 weeks of treatment with Huáng qí and its complex (Figures 1A,B) and were significantly lower (*p* < 0.05) than the control group. The Huáng qí and its complex reduced the MDA levels in the brains of SAMP8 mice for 8 consecutive weeks. Xu et al. (5) reported that administering spermidine, spermine, and rapamycin (0.78 mg/kg/d) for 8 consecutive weeks alleviated OS in the SAMP8 mouse brain by decreasing MDA levels and increasing brain SOD activity, which agreed with the present study results.

**Figure 2:**
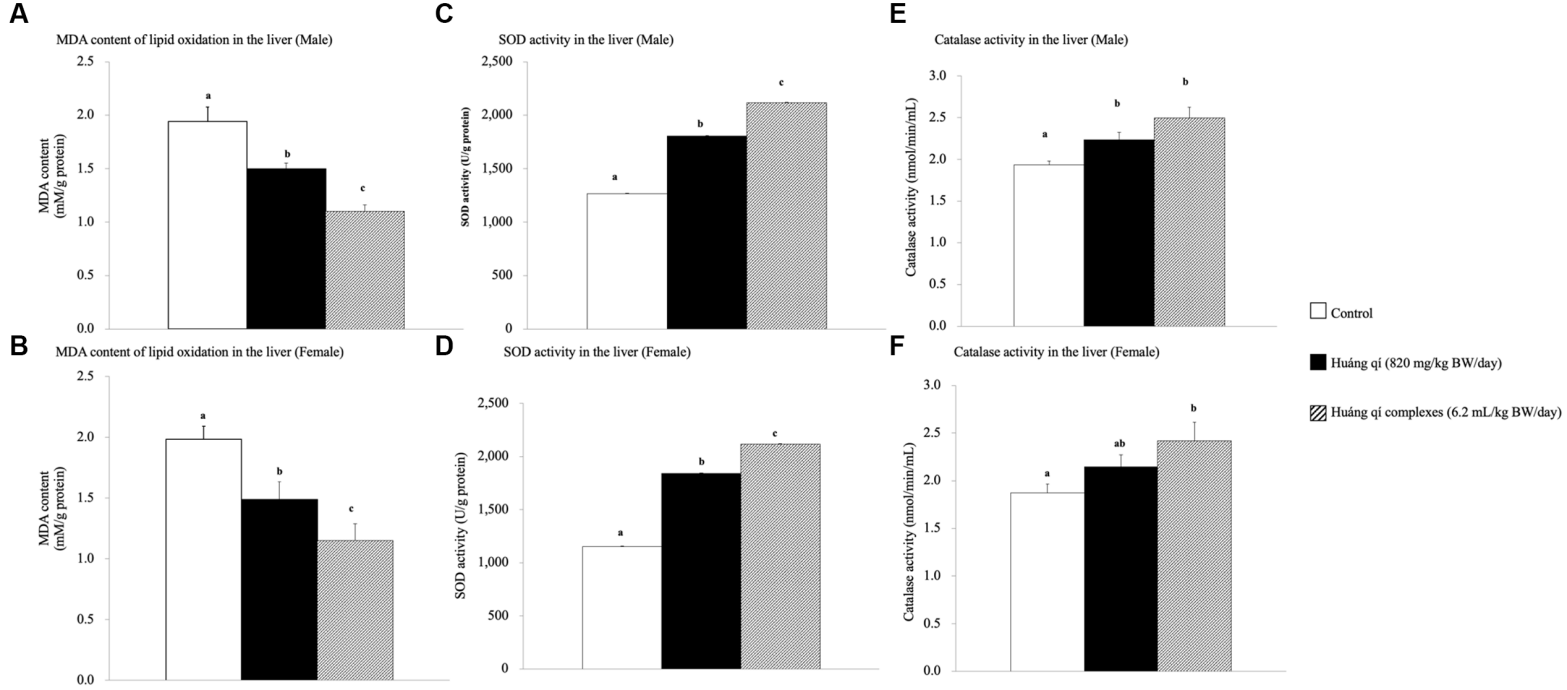
Effects of a continuous 8-weeks of tube feeding Huáng qí and its complex on SAMP8 mice (*n* = 8) malondialdehyde (MDA) content **(A,B)**, superoxide dismutase (SOD) activity **(C,D)**, and catalase (CAT) activity **(E,F)** in the liver. Different lowercase letters represent significant differences (*p* < 0.05).

Continuous feeding of Huáng qí and its complex for 8 weeks resulted in a significant decrease in MDA contents in the livers of male and female mice compared to the control group (*p* < 0.05), and the effects were better in the groups fed the Huáng qí complex (Figures 2A,B). However, the implication was that administering either Huáng qí or its complex for 8 weeks reduced MDA levels in the liver of mice. The Huáng qí compounds reduced MDA levels the most in the liver, and the effect was better in males than females. ROS damaged the neuronal membrane components and are associated with the pathophysiology of neurological diseases (age -related), such as SAMP8 mice and AD, where mice have altered free radicals, and SOD activity and MDA levels increased, while glutathione levels (in the liver or brain) decreased (13, 55, 76–78). In addition, several reports have indicated that MDA may contribute to producing highly immunogenic MDA acetaldehyde compounds under sustained OS. These compounds participate in secondary reactions by promoting intra- or inter-molecular protein/DNA cross-linking and chronic inflammation, disease, aging, and DNA damage (15, 73, 79–83).

#### 3.7.2. Superoxide dismutase in the liver

Superoxide dismutase (SOD) is part of the primary defense system that scavenges free radicals to reduce OS (5). Damage caused by OS leads to neurodegenerative diseases, and *in vivo,* antioxidant capacity affects memory capacity (70, 84). This study showed that liver SOD activity increased significantly in the experimental groups (*p* < 0.05) compared to the control group after feeding Huáng qí and its complex for 8 consecutive weeks (Figures 2C,D). Thus, supplementing male and female mice with the Huáng qí complex enhanced SOD activity.

#### 3.7.3. Catalase activity in the liver

Significant increases in liver CAT activity were detected in all male and female experimental groups compared to the control group after feeding male and female mice Huáng qí and its complex for 8 weeks (Figures 2E,F). Ornoy et al. (50) reported that valproic acid (VPA) and S-adenosylmethionine (SAM, with saline) increase SOD and CAT activities, respectively. However, a post -VPA injection of SAM prevented autistic -like behavior in mice, suggesting that the *in vivo* antioxidant capacity originated from the nutritional supplement while effectively mitigating oxidative damage in the brain exposed to OS. Alternatively, severe oxidative damage (liver, micronucleus, and DNA) occurs due to decreased antioxidant level/activity (85). These results indicate that continuously feeding either Huáng qí or its complex for 8 weeks reduced OS in SAMP8 mice *in vivo* (brain and liver).

## 4. Conclusion

This study showed that Huáng qí and its complex reduced aging and improved learning and memory in the SAMP8 mouse model after continuous supplementation for 8 weeks. In addition, the enhanced SOD and CAT activities in the liver and reduced MDA content to achieve antioxidant effects may have reduced oxidative damage (liver and brain), thus promoting anti-aging effects. Therefore, Huáng qí and its complex may be more effective for age-related brain impairment than conventional agents as a novel nutritional intervention or as a nutritional supplement with full nutritional potential for cognitive health. Overall, Huáng qí is a safe and effective herbal supplement targeting various pathologies. In particular, APS is a valuable immunomodulatory agent, but further studies are necessary to substantiate clinical efficacy in humans.

## Data availability statement

The original contributions presented in the study are included in the article/Supplementary files, further inquiries can be directed to the corresponding authors.

## Ethics statement

The animal study was reviewed and approved by the Committee on Animal Research, Providence University, under code 220191211 A008 approved the protocol.

## Author contributions

M-YC, Y-CW, and S-YW: conceptualization. C-HC, T-HW, and M-JH: data curation and methodology. P-HH, Y-CW, and S-YW: investigation. P-HH, P-HL, and M-FW: writing —original draft. P-HL and M-FW: writing—review and editing. All authors have read andagreed to the published version of the manuscript.

## Funding

The authors declare that this study received funding from PhytoHealth Co. The funder was not involved in the study design, collection, analysis, interpretation of data, the writing of this article, or the decision to submit it for publication.

## Conflict of interest

T-HW was employed by PhytoHealth Co.

The remaining authors declare that the research was conducted in the absence of any commercial or financial relationships that could be construed as a potential conflict of interest.

## Publisher’s note

All claims expressed in this article are solely those of the authors and do not necessarily represent those of their affiliated organizations, or those of the publisher, the editors and the reviewers. Any product that may be evaluated in this article, or claim that may be made by its manufacturer, is not guaranteed or endorsed by the publisher.
