# Supplementary material for: Potential antidepressant effects of a dietary supplement from Huáng qí and its complex in aged senescence-accelerated mouse prone-8 mice
Source: Front Nutr. 2023 Jul 28;10:1235780. doi: 10.3389/fnut.2023.1235780 (PMC10421658; doi:10.3389/fnut.2023.1235780)
Supplement: Supplementary file 1 [file Data_Sheet_1.PDF]

Attached Table. The scoring evaluation form

Item	Define	Grade 0	Grade 1	Grade 2	Grade 3	Grade 4
Behavior<br><br>Reactivity	The most intensive exploratory response observed within 30 seconds.	Natural behavior.	A. Abnormal gait with no lessening of agility and behavior patterns.<br><br>B. Restlessness.	Definite decrease in agility and behavior patterns.	Does not move voluntarily but will move if nudged.	Immobile.
Passivity	Escape reaction from pinching of the nuchal skin or from hanging by the forelimb.	Natural escape reaction to plnching.	Decrease in escape reaction to plnching	Loss of escape reaction to pinching. Preserved righting reaction to manual turn over.	Neither escapes reaction to pinching not righting reaction. Escape reaction to hanging by the forelimb.	Escape reaction nil.

**II) Appearance**<br>**1. Skin and hair**<br>**(1) Glossiness**	**Glossinss.**	**Natural gloss.**	**Decrease in gloss.**	**Complete disappearance of gloss.**	**Complete disappearance of gloss and hair appears dirty.**	**Complete disappearance of gloss and hair looks very dirty.**
**Coarseness**	**Coarseness of hair on the head, nucha and dorsum determined according to the number of palpable, fine clumps of hair**	**No coarseness.**	**Coarseness of less than an area of the head.**	**Coarseness of less than doubles the area of the head.**	**Coarseness of less than 3 times areas of the head.**	**Complete disappearance of gloss and hair looks very dirty.**
**Loss of hair**	**Loss or thinning of hair on the head, nucha and head, nucha and dorsum except for changes due to ulcer or periophthalmic**	**Neither loss nor thinning of hair.**	**A. Loss of hair in less than an area of the head.**	**A. Loss of hair in over one area of the head, less than in 1/4 of total area.**	**Loss of hair in more than 1/4, in less than 1/2 of total area.**	**Loss of hair in over 1/2 of total area.**

	lesions.		B. Thinning of hair in less than 1/2 of the area.	B. Thinning of hair in more than 1/2 of total area.		
Skin ulcers	Ulcer or healed ulcer on entire skin except for changes associated with periophthalmic lesions.	No evidence of ulcer.	Healed ulcer or ulcer with scab.	Ulcer without healing tendency, in less than one area of the head.	Ulcer without healing tendency in more than one area of the head, in less than 1/4 area of all the skin.	Ulcer without healing tendency in more than 1/4 area of whole skin.
Eyes<br><br>(1)Periophthalmic Lesions	Catarrhal changes in the periophthalmic area or swelling of the palpebra.	No changes.	Catarrhal changes limited to periophthalmic area or swelling of palpebra.	Catarrhal changes extending to nose.	Catarrhal changes extending further.	
(2)Corneal pacity	Opaque changes of cornea with rough surface by direct ophthalmoscopy.	No opacity.	Opacity with visible iris.	Opacity with visible iris.<br><br>Positive retinal reflex by transillumination.	Opacity of entire cornea.	

**(3)Ulcer of the cornes**	**Opaque changes of cornea with rough surface by direct ophthalmoscopy.**	**No ulcer**	**Linear ulcer corresponding to palpebral fissure.**	**Extension of ulcer over most of the area.**	**Ulcer of entire cornea.**	
**(4) Cataract**	**Opaque changes of crystalline lens without retinal reflex by transillumination.**<br><br>**Impossible to soccer because of coexistence of grade 3 corneal opacity or ulcer.**	**Natural reflection.**	**Diminished reflection.**	**No reflection.**		

**Spine**<br><br>**(1)Lordokyphosis of the spine**	**Examined by inspection and palpation.**	**Natural anteroposterior curvature.**	**Increased curvature disappears with digital pressure on the dorsum.**	**Increased curvature disappears with a combination of manual cephalocaudal traction and digital pressure on the dorsum.**	**Permanent curvature.**	

Attached Table. The scoring evaluation form

Score	0	1	2	3	4
**Behavior**					
Reacivity					
Passivity					
**Skin**					
Glossiness					
Coarseness					
Hair loss					
Ulcer					
**Eyes**					
Periophthalmic lesion					
**Spine**					
Lordokyphosis					
Total					
